# Methylation-derived inflammatory measures and lung cancer risk and survival

**DOI:** 10.1186/s13148-021-01214-2

**Published:** 2021-12-16

**Authors:** Naisi Zhao, Mengyuan Ruan, Devin C. Koestler, Jiayun Lu, Lucas A. Salas, Karl T. Kelsey, Elizabeth A. Platz, Dominique S. Michaud

**Affiliations:** 1 Department of Public Health and Community Medicine, Tufts University School of Medicine, Tufts University, 136 Harrison Avenue, Boston, MA 02111 USA; 2 Department of Biostatistics and Data Science, Medical Center, University of Kansas, Kansas City, KS USA; 3 University of Kansas Cancer Center, Kansas City, KS USA; 4 Department of Epidemiology, Johns Hopkins Bloomberg School of Public Health, Baltimore, MD USA; 5 The Sidney Kimmel Comprehensive Cancer Center at Johns Hopkins, Baltimore, MD USA; 6 Department of Epidemiology, Geisel School of Medicine, Dartmouth College, Lebanon, NH USA; 7 Department of Epidemiology, Brown University, Providence, RI USA; 8 Department of Pathology and Laboratory Medicine, Brown University, Providence, RI USA

**Keywords:** Lung cancer, DNA methylation, Methylation-based inflammation measures, C-reactive protein, mdNLR

## Abstract

**Background:**

Examining immunity-related DNA methylation alterations in blood could help elucidate the role of the immune response in lung cancer etiology and aid in discovering factors that are key to lung cancer development and progression. In a nested, matched case–control study, we estimated methylation-derived NLR (mdNLR) and quantified DNA methylation levels at loci previously linked with circulating concentrations of C-reactive protein (CRP). We examined associations between these measures and lung cancer risk and survival.

**Results:**

Using conditional logistic regression and further adjusting for BMI, batch effects, and a smoking-based methylation score, we observed a 47% increased risk of non-small cell lung cancer (NSCLC) for one standard deviation (SD) increase in mdNLR (*n* = 150 pairs; OR: 1.47, 95% CI 1.08, 2.02). Using a similar model, the estimated CRP Scores were inversely associated with risk of NSCLC (e.g., Score 1 OR: 0.57, 95% CI: 0.40, 0.81). Using Cox proportional hazards models adjusting for age, sex, smoking status, methylation-predicted pack-years, BMI, batch effect, and stage, we observed a 28% increased risk of dying from lung cancer (*n* = 145 deaths in 205 cases; HR: 1.28, 95% CI: 1.09, 1.50) for one SD increase in mdNLR.

**Conclusions:**

Our study demonstrates that immunity status measured with DNA methylation markers is associated with lung cancer a decade or more prior to cancer diagnosis. A better understanding of immunity-associated methylation-based biomarkers in lung cancer development could provide insight into critical pathways.

**Supplementary Information:**

The online version contains supplementary material available at 10.1186/s13148-021-01214-2.

## Background

Lung cancer is the leading cause of cancer death in the USA, projected to account for 21.7% of all cancer deaths in 2021 [1]. A large percentage of lung cancer patients are diagnosed at an advanced stage [2] and five-year relative survival rates for those patients are between 3 and 6% [3]. Thus, early detection remains a key strategy to improve survival. However, the currently recommended strategy for lung cancer screening—low-dose computed tomography (LDCT) for persons 50 to 80 years old with at least a 20 pack-year smoking history and currently smoke or have quit within the past 15 years—is expensive and has a high false positive rate [4, 5]. Modifying the current lung cancer screening strategy by performing risk stratification could help prioritize LDCT screening and optimize secondary prevention. We propose that immune system markers could be incorporated into such risk stratification tools to help identify persons at higher risk of lung cancer to target for screening.

While smoking is the most important risk factor for lung cancer in the population, there is growing evidence that the immune system, in response to or independent of smoking, plays an important role in lung cancer development, acting potentially through the genesis of chronic inflammation [6]. For instance, an aggregated genome-wide association studies (GWASs) analysis of lung cancer risk found a direct causal effect of BMI on small cell lung cancer and an inverse effect on lung adenocarcinoma, suggesting the complexity of the role BMI and chronic inflammation plays in lung cancer subtypes [7]. Furthermore, it is plausible that inflammatory profiles prior to lung cancer diagnosis are associated with lung cancer-specific survival. Markers of systemic inflammation, including elevated levels of C-reactive protein (CRP) and the peripheral blood neutrophil-to-lymphocyte ratio (NLR), also have been identified as robust markers of cancer-associated inflammation [8, 9]. Elevated CRP levels [8], elevated serum levels of pro-inflammatory cytokines [10–12], increased neutrophil counts and decreased lymphocyte counts [13, 14], and polymorphisms in inflammation-related genes [15–18] have been associated with increased lung cancer risk. These inflammatory measures have also been associated with poor survival of lung cancer patients in several retrospective and a few prospective studies [19–21]. In addition, both experimental and epidemiologic studies support a role for chronic inflammation as a hallmark of cancer development and progression [8, 22–25]. We posit that a better understanding of the role of inflammation in lung cancer etiology could be gained by examining DNA methylation alterations in blood that are associated with the systemic immune response.

In the current study, we first predicted peripheral blood leukocyte composition and a neutrophil to lymphocyte index using validated DNA methylation markers (mdNLR), then quantified DNA methylation levels at loci previously linked with circulating concentrations of CRP, and calculated methylation-derived immune cell ratios by using an expanded deconvolution library. We evaluated the associations of these potential markers with lung cancer risk and lung cancer-specific survival. To address this question, we used pre-diagnostic blood samples of cases and controls obtained from the CLUE I/II cohorts. Our analyses controlled for self-reported smoking and methylation-predicted cumulative smoking in order to better focus our examinations on the DNA methylation marks that are informative of the immune response profile [26].

## Results

### Population characteristics

Characteristics of the 208 lung cancer cases and their 208 matched controls included in this analysis are presented in Table 1. Over 99% of the majority of participants were White. The median time between blood draw and lung cancer diagnosis was 14 years. The median age at blood draw in 1989 was 59 and 57 years in cases and controls, respectively. Overall, 55% of cases and controls were women and 11% were never smokers (Table 1).

**Table 1:** Baseline characteristics of lung cancer cases and matched controls nested in CLUE I/II

	Controls	Cases		
		All lung cancers	Non-small cell lung cancer	Small cell lung cancer
*N*	208	208	150	29
Median age (range, years)	56 [28, 81]	59 [30, 83]	58 [36, 83]	53 [30, 71]
Median time before diagnosis (range, years)		14 [0, 29]	14 [0, 28]	12 [2, 29]
Sex				
Male	95 (45.7%)	95 (45.7%)	61 (40.7%)	16 (55.2%)
Female	113 (54.3%)	113 (54.3%)	89 (59.3%)	13 (44.8%)
Race				
White	208 (100%)	205 (98.6%)	149 (99.3%)	28 (96.6%)
Black	0 (0%)	3 (1.4%)	1 (0.7%)	1 (3.4%)
Cigarette smoking status				
Never smoker	22 (10.6%)	22 (10.6%)	15 (10.0%)	1 (3.4%)
Ever smoker	80 (38.5%)	80 (38.5%)	56 (37.3%)	10 (34.5%)
Current smoker	106 (51.0%)	106 (51.0%)	79 (52.7%)	18 (62.1%)
Median cigarette smoking Intensity (range, cig/day)	20 [0, 80]	20 [0, 80]	20 [0, 70]	20 [0, 80]
Cigar or pipe smoking				
Never	176 (84.6%)	178 (85.6%)	132 (88.0%)	25 (86.2%)
Ever	28 (13.5%)	26 (12.5%)	15 (10.0%)	4 (13.8%)
Current	4 (1.9%)	4 (1.9%)	3 (2.0%)	0 (0%)
Median BMI, kg/m^2^ (range)	25.8 [18.0, 40.9]	25.6 [15.5, 42.6]	25.4 [15.5, 42.6]	25.6 [18.3, 34.7]
mdNLR mean (SD)	1.74 (0.976)	1.86 (1.32)	1.85 (1.19)	1.65 (1.07)
mdNLR median (range)	1.58 [0.13, 6.42]	1.59 [0.28, 11.90]	1.56 [0.28, 9.45]	1.44 [0.39, 4.81]
CRP score 1 mean (SD)	0.02 (1.01)	− 0.02 (0.99)	− 0.04 (0.97)	0.47 (1.08)
CRP score 1 median (range)	0.06 [− 2.42, 2.99]	− 0.03 [− 3.26, 2.94]	− 0.03 [− 3.26, 2.94]	0.41 [− 1.93, 2.16]
CRP score 2 mean (SD)	0.04 (1.03)	− 0.04 (0.98)	− 0.06 (0.95)	0.36 (1.17)
CRP score 2 median (range)	0.01 [− 3.35, 3.93]	− 0.10 [− 2.68, 3.10]	− 0.11 [− 2.68, 3.10]	0.24 [− 2.34, 3.10]
CRP score 3 mean (SD)	− 0.06 (0.98)	0.06 (1.02)	0.09 (1.03)	0.32 (0.97)
CRP score 3 median (range)	− 0.09 [− 2.43, 2.51]	0.08 [− 2.34, 2.95]	0.10 [− 2.34, 2.95]	0.38 [− 2.19, 1.97]

### Methylation-derived mdNLR index, leukocyte proportions, and lung cancer risk

We observed a 47% increased risk of non-small cell lung cancer (NSCLC) for one standard deviation increase in mdNLR (*n* = 150 pairs; OR: 1.47 [1.08, 2.02]). However, higher mdNLR values were not statistically associated with overall risk of lung cancer in our study. This association was comparable for NSCLC cases diagnosed within 10 years and beyond 10 years after blood draw. No stable associations could be estimated for small cell lung cancer (SCLC). After multiple comparison adjustments, monocyte/lymphocyte ratio showed a borderline significant 65% increased risk of NSCLC for each standard deviation increase (*n* = 150 pairs; OR: 1.65, adjusted CI: [0.99, 2.76]). In addition, immune cell ratios for CD4/CD8, NLR, B cell/lymphocyte, T cell/lymphocyte, Neu + Mono/lymphocyte, Eos/lymphocyte, CD4nv/lymphocyte, B cell/CD8, CD8/Treg, Bnv/Bmem, CD4nv/CD4mem, CD8nv/CD8mem, and Treg > 0 vs. Treg = 0 were not statistically significantly associated with lung cancer risk overall or by histologic types (Table 2).

**Table 2:** Association between methylation-predicted immune cell profiles and risk of total lung cancer and NSCLC risk, overall and stratified by time to diagnosis, case–control study nested in the CLUE I/II cohort

	All lung cancers^a^ OR (95% CI)	Non-small cell lung cancer^b^ OR (95% CI)
mdNLR^c^	1.11 (0.89, 1.38)	**1.47 (1.08, 2.02)**
Time to diagnosis ≤ 10 years	0.97 (0.69, 1.35)	1.41 (0.90, 2.20)
Time to diagnosis > 10 years	1.22 (0.91, 1.64)	1.57 (0.98, 2.49)
CD4/CD8 Ratio^c^	1.17 (0.80, 1.72)	1.16 (0.74, 1.82)
Time to diagnosis ≤ 10 years	0.94 (0.56, 1.57)	0.79 (0.38, 1.66)
Time to diagnosis > 10 years	1.47 (0.82, 2.62)	1.72 (0.77, 3.85)
B cell/lymphocyte ratio^c^	1.07 (0.73, 1.57)	0.94 (0.58, 1.52)
Time to diagnosis ≤ 10 years	1.16 (0.63, 2.14)	0.91 (0.42, 1.97)
Time to diagnosis > 10 years	0.98 (0.60, 1.59)	0.93 (0.50, 1.71)
T cell/lymphocyte ratio^c^	1.06 (0.72, 1.56)	0.94 (0.60, 1.48)
Time to diagnosis ≤ 10 years	1.11 (0.62, 1.98)	1.03 (0.49, 2.16)
Time to diagnosis > 10 years	1.05 (0.63, 1.76)	0.88 (0.48, 1.62)
Monocyte/lymphocyte ratio^c^	1.15 (0.78, 1.69)	1.65 (0.99, 2.76)
Time to diagnosis ≤ 10 years	1.18 (0.66, 2.11)	1.62 (0.75, 3.51)
Time to diagnosis > 10 years	1.16 (0.69, 1.94)	1.81 (0.86, 3.80)
(Neutrophil + monocyte)/lymphocyte ratio^c^	1.13 (0.79, 1.61)	1.46 (0.87, 2.44)
Time to diagnosis ≤ 10 years	0.96 (0.59, 1.56)	1.32 (0.67, 2.60)
Time to diagnosis > 10 years	1.26 (0.78, 2.04)	1.62 (0.80, 3.29)
Eosinophil/lymphocyte ratio^c^	0.94 (0.64, 1.38)	0.89 (0.53, 1.49)
Time to diagnosis ≤ 10 years	0.81 (0.44, 1.49)	1.21 (0.48, 3.08)
Time to diagnosis > 10 years	1.01 (0.64, 1.59)	0.73 (0.36, 1.48)
CD4 naïve/lymphocyte ratio^c^	1.22 (0.83, 1.80)	1.36 (0.84, 2.20)
Time to diagnosis ≤ 10 years	1.47 (0.72, 2.99)	1.70 (0.74, 3.93)
Time to diagnosis > 10 years	1.11 (0.68, 1.80)	1.19 (0.67, 2.12)
B cell/CD8 ratio^c^	0.97 (0.68, 1.38)	1.00 (0.66, 1.52)
Time to diagnosis ≤ 10 years	1.06 (0.61, 1.83)	1.11 (0.58, 2.11)
Time to diagnosis > 10 years	0.85 (0.45, 1.62)	0.90 (0.44, 1.83)
CD8/treg ratio^c^	0.81 (0.55, 1.19)	0.73 (0.45, 1.18)
Time to diagnosis ≤ 10 years	0.86 (0.47, 1.59)	0.72 (0.33, 1.56)
Time to diagnosis > 10 years	0.78 (0.45, 1.35)	0.68 (0.33, 1.38)
Treg > 0 versus Treg = 0^c^	1.05 (0.74, 1.50)	1.07 (0.70, 1.63)
Time to diagnosis ≤ 10 years	0.95 (0.53, 1.70)	1.12 (0.55, 2.27)
Time to diagnosis > 10 years	1.10 (0.70, 1.73)	1.06 (0.61, 1.83)
B cell naïve/B cell memory ratio^c^	0.85 (0.58, 1.25)	0.87 (0.57, 1.32)
Time to diagnosis ≤ 10 years	0.65 (0.24, 1.76)	0.65 (0.21, 2.01)
Time to diagnosis > 10 years	0.93 (0.56, 1.56)	0.96 (0.56, 1.66)
CD4 naïve/CD4 memory ratio^c^	1.04 (0.73, 1.48)	1.59 (0.81, 3.13)
Time to diagnosis ≤ 10 years	0.98 (0.59, 1.64)	2.67 (0.65, 11.01)
Time to diagnosis > 10 years	1.18 (0.60, 2.32)	1.34 (0.58, 3.10)
CD8 naïve/CD8 memory ratio^c^	0.99 (0.72, 1.37)	1.01 (0.53, 1.92)
Time to diagnosis ≤ 10 years	0.66 (0.15, 2.81)	0.74 (0.17, 3.15)
Time to diagnosis > 10 years	1.04 (0.71, 1.53)	1.73 (0.36, 8.38)

Bold OR and CI values indicate statistical significance. Underline OR and CI values indicate borderline statistical significance after multiple comparison adjustment

^a^208 cases and 208 controls matched on age at blood draw, sex, smoking status, and model further adjusting for BMI, surrogate variables for batch effects, and a methylation-predicted pack-years smoked

^b^150 cases and 150 controls matched on age at blood draw, sex, smoking status, and model further adjusting for BMI, surrogate variables for batch effects, and a methylation-predicted pack-years smoked

^c^OR results reported per 1 unit SD increase (mdNLR SD: 1.16; CD4/CD8 ratio SD: 2.07; BL ratio SD: 0.0526; TL ratio SD: 0.0802; ML ratio SD: 0.118; NM/L ratio SD: 1.22; EL ratio SD: 0.0722; CD4nv/L ratio SD: 0.0915; B/CD8 ratio SD: 3.07; CD8/Treg ratio SD: 115; Bnv/Bmem ratio SD: 109; CD4nv/CD4mem ratio SD: 0.429; CD8nv/CD8mem ratio SD: 2.05) and OR results reported for Treg > 0 versus 0; Bonferroni-adjusted CI reported for all except mdNLR (a prior hypothesis)

### Methylation-derived CRP scores and lung cancer risk

CRP Score 1 was built using 54 CpG sites that were previously associated with inflammatory markers, while CRP Score 2 and 3 were each built with a subset of these 54 CpGs that were putative cell-specific or cell type invariant, respectively. Using data from a previously published pancreatic cancer dataset [27], all three scores were moderately correlated with log CRP and log IL-6 levels (Table 3). In this nested case–control study, we found all three CRP Scores inversely associated with risk of NSCLC after additionally adjusting for methylation-predicted pack-years (*n* = 150 pairs; Score 1 OR: 0.57 [0.40, 0.81]; Score 2 OR: 0.62 [0.45, 0.84]; Score 3 OR: 0.65 [0.44, 0.95]). We also found statistically significant inverse association between CRP Score 1 and risk of NSCLC among cases diagnosed within 10 years and beyond 10 years, and between CRP Score 2 for NSCLC cases diagnosed within 10 years of blood draw (Table 4). CRP Scores 1, 2, and 3 were not associated with lung cancer risk when taking into account the matching factors and only adjusting for BMI and four surrogate variables for batch effects (*n* = 208 pairs; Score 1 OR: 0.96 [0.77, 1.21]; Score 2 OR: 0.89 [0.71, 1.11]; Score 3 OR: 1.11 [0.89, 1.40]). However, when additionally adjusting for methylation-predicted pack-years, inverse associations with total lung cancer risk were observed for Score 1 (OR: 0.76 [0.59, 0.99]) and Score 2 (OR: 0.77 [0.61, 0.98]). We also observed a 33% decreased risk of lung cancer for one standard deviation increase in CRP Score 1 (OR: 0.67 [0.47, 0.97]) among those with time to diagnosis over 10 years.

**Table 3:** Correlations between methylation-based CRP scores and circulating log-CRP level, log-IL6 level, peripheral blood leukocyte types, BMI, and smoking score residual among controls only

	Score 1^b^	Score 2^c^	Score 3^d^
	Spearman (*p *value)	Spearman (*p* value)	Spearman (*p* value)
Pancreatic cancer study (controls only)			
Log-CRP level^a^	0.284 (4.27e−06)	0.247 (6.96e−05)	0.297 (1.56e−06)
Log-IL6 level^a^	0.213 (7.53e−04)	0.158 (1.32e−02)	0.203 (1.33e−03)
CLUE I/II (controls only)			
CD4T	0.425 (1.65e−10)	0.359 (1.00e−07)	0.212 (2.13e−03)
CD8T	0.504 (8.78e−15)	0.461 (2.34e−12)	0.390 (5.74e−09)
NK	0.062 (3.70e−01)	0.175 (1.17e−02)	0.026 (7.09e−01)
B cell	0.103 (1.38e−01)	0.069 (3.23e−01)	− 0.027 (6.99e−01)
Neutrophils	− 0.467 (1.19e−12)	− 0.422 (2.24e−10)	− 0.264 (1.17e−04)
Monocytes	− 0.292 (1.92e−05)	− 0.330 (1.17e−06)	− 0.288 (2.38e−05)
mdNLR	− 0.488 (7.66e−14)	− 0.449 (1.01e−11)	− 0.281 (3.96e−05)
BMI	0.025 (7.22e−01)	0.070 (3.12e−01)	− 0.063 (3.68e−01)
Methylation-predicted pack-years residual^e^	0.475 (4.46e−13)	0.375 (2.43e−08)	0.632 (1.42e−24)

^a^Correlations with log-CRP level and log-IL6 level were tested with a pancreatic cancer dataset[43]

^b^CpG Score 1 is built using 54 CpG sites

^c^CpG Score 2 is built using the top 10 highly cell-specific CpG sites

^d^CpG Score 3 is built using the 10 modestly cell-specific CpG sites

^e^We calculated a pack-years methylation score to represent pack-years smoked associated methylation alterations. This score correlates with gene expression changes that are affected by smoking

**Table 4:** Association between methylation-based CRP scores and risk of total lung cancer and NSCLC risk, overall and stratified by time to diagnosis, CLUE I/II cohort

	All lung cancers^a^ OR (95% CI)	Non-small cell lung cancer^b^ OR (95% CI)
CRP Score 1^c^	**0.76 (0.59, 0.99)**	**0.57 (0.40, 0.81)**
Time to diagnosis ≤ 10 years	0.84 (0.56, 1.26)	**0.57 (0.33, 0.97)**
Time to diagnosis > 10 years	**0.67 (0.47, 0.97)**	**0.53 (0.32, 0.88)**
CRP Score 2^d^	**0.77 (0.61, 0.98)**	**0.62 (0.45, 0.84)**
Time to diagnosis ≤ 10 years	0.79 (0.54, 1.17)	**0.49 (0.28, 0.85)**
Time to diagnosis > 10 years	0.73 (0.53, 1.02)	0.68 (0.46, 1.00)
CRP Score 3^e^	0.79 (0.58, 1.07)	**0.65 (0.44, 0.95)**
Time to diagnosis ≤ 10 years	0.87 (0.53, 1.42)	0.64 (0.35, 1.18)
Time to diagnosis > 10 years	0.73 (0.49, 1.09)	0.62 (0.36, 1.05)

Bold OR and CI values indicate statistical significance

^a^208 cases and 208 controls matched for age at blood draw, sex, smoking status, and model further adjusting for BMI, surrogate variables for batch effects, and a methylation-predicted pack-years smoked

^b^150 cases and 150 controls matched for age at blood draw, sex, smoking status, and model further adjusting for BMI, surrogate variables for batch effects, and a methylation-predicted pack-years smoked. The matched pairs were kept within each stratum

^c^CpG Score 1 is built using 54 CpG sites and OR results reported per 1 unit of SD of CpG Score 1 (SD 1)

^d^CpG Score 2 is built using the top 10 highly cell-specific CpG sites and OR results reported per 1 unit of SD of CpG Score 2 (SD 1)

^e^CpG Score 3 is built using the 10 modestly cell-specific CpG sites and OR results reported per 1 unit of SD of CpG Score 3 (SD 1)

### Survival analysis

**Fig. 1:**
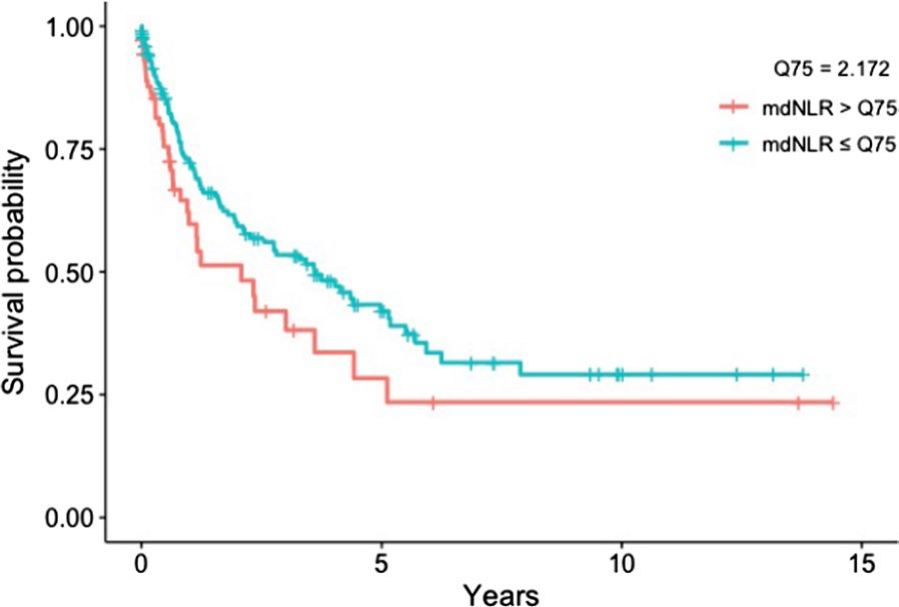
Survival curves for lung cancer-specific mortality among lung cancer cases in the mdNLR high and low groups (> or ≤ 75% quartiles). Plot adjusted for age, sex, smoking status, methylation-predicted pack-years smoked, BMI, stage, and batch effects

We examined whether the mdNLR, methylation-derived immune cell ratios, and CRP Scores were associated with risk of dying of lung cancer among lung cancer cases (Table 5, Fig. 1).

**Table 5:** Association between immune cell ratios and methylation-based CRP scores and lung cancer-specific mortality among lung cancer cases, CLUE I/II cohort

	All lung cancers^a^ HR (95% CI)	Non-small cell lung cancer^b^ HR (95% CI)
mdNLR^c^	**1.28 (1.09, 1.50)**	**1.47 (1.20, 1.81)**
Time to diagnosis ≤ 10 years	**1.34 (1.01, 1.76)**	**1.73 (1.19, 2.51)**
Time to diagnosis > 10 years	1.20 (0.98, 1.48)	**1.39 (1.05, 1.85)**
CD4/CD8 ratio^c^	1.02 (0.76, 1.36)	1.07 (0.78, 1.48)
Time to diagnosis ≤ 10 years	1.28 (0.79, 2.07)	1.29 (0.70, 2.38)
Time to diagnosis > 10 years	0.96 (0.65, 1.41)	1.03 (0.66, 1.62)
B cell/lymphocyte ratio^c^	0.97 (0.73, 1.30)	1.06 (0.74, 1.51)
Time to diagnosis ≤ 10 years	0.87 (0.50, 1.50)	0.99 (0.52, 1.89)
Time to diagnosis > 10 years	1.11 (0.75, 1.63)	1.18 (0.75, 1.85)
T cell/lymphocyte ratio^c^	1.01 (0.78, 1.31)	0.95 (0.69, 1.31)
Time to diagnosis ≤ 10 years	1.01 (0.62, 1.64)	0.94 (0.53, 1.68)
Time to diagnosis > 10 years	1.05 (0.69, 1.60)	0.91 (0.54, 1.52)
Monocyte/Lymphocyte Ratio^c^	1.16 (0.87, 1.55)	1.21 (0.85, 1.72)
Time to diagnosis ≤ 10 years	1.45 (0.87, 2.43)	1.52 (0.80, 2.89)
Time to diagnosis > 10 years	1.25 (0.80, 1.96)	1.54 (0.84, 2.84)
(Neutrophil + monocyte)/lymphocyte ratio^c^	1.29 (1.00, 1.67)	**1.48 (1.04, 2.11)**
Time to diagnosis ≤ 10 years	1.36 (0.87, 2.13)	1.76 (0.95, 3.24)
Time to diagnosis > 10 years	1.22 (0.86, 1.74)	1.41 (0.87, 2.29)
Eosinophil/lymphocyte ratio^c^	1.01 (0.76, 1.35)	0.86 (0.60, 1.23)
Time to diagnosis ≤ 10 years	1.55 (0.93, 2.59)	1.40 (0.74, 2.67)
Time to diagnosis > 10 years	0.98 (0.67, 1.44)	0.77 (0.49, 1.21)
CD4 naïve/lymphocyte ratio^c^	0.92 (0.67, 1.27)	0.95 (0.65, 1.40)
Time to diagnosis ≤ 10 years	0.92 (0.53, 1.59)	0.73 (0.35, 1.53)
Time to diagnosis > 10 years	0.91 (0.60, 1.38)	0.99 (0.61, 1.60)
B cell/CD8 ratio^c^	1.03 (0.82, 1.29)	1.07 (0.83, 1.38)
Time to diagnosis ≤ 10 years	1.01 (0.69, 1.49)	1.04 (0.64, 1.69)
Time to diagnosis > 10 years	1.05 (0.71, 1.55)	1.20 (0.74, 1.95)
CD8/treg ratio^c^	0.92 (0.67, 1.27)	0.92 (0.63, 1.35)
Time to diagnosis ≤ 10 years	0.73 (0.42, 1.26)	0.63 (0.30, 1.32)
Time to diagnosis > 10 years	1.03 (0.66, 1.62)	1.09 (0.63, 1.88)
Treg > 0 versus treg = 0^c^	0.96 (0.72, 1.28)	0.91 (0.64, 1.30)
Time to diagnosis ≤ 10 years	1.27 (0.76, 2.13)	1.37 (0.70, 2.69)
Time to diagnosis > 10 years	0.85 (0.58, 1.25)	0.75 (0.45, 1.26)
B cell naïve/B cell memory Ratio^c^	1.09 (0.84, 1.41)	1.23 (0.89, 1.70)
Time to diagnosis ≤ 10 years	1.31 (0.86, 1.99)	1.66 (0.74, 3.71)
Time to diagnosis > 10 years	1.03 (0.75, 1.42)	1.19 (0.73, 1.93)
CD4 naïve/CD4 memory ratio^c^	0.97 (0.70, 1.34)	1.03 (0.70, 1.52)
Time to diagnosis ≤ 10 years	0.91 (0.53, 1.57)	0.71 (0.32, 1.59)
Time to diagnosis > 10 years	1.00 (0.64, 1.57)	1.17 (0.70, 1.96)
CD8 naïve/CD8 memory ratio^c^	0.96 (0.70, 1.32)	1.10 (0.77, 1.57)
Time to diagnosis ≤ 10 years	1.09 (0.63, 1.88)	1.19 (0.59, 2.42)
Time to diagnosis > 10 years	0.92 (0.61, 1.40)	1.05 (0.67, 1.65)
CRP Score 1^cd^	0.92 (0.67, 1.26)	0.88 (0.60, 1.30)
Time to diagnosis ≤ 10 years	1.00 (0.57, 1.75)	1.22 (0.67, 2.22)
Time to diagnosis > 10 years	1.04 (0.67, 1.63)	0.82 (0.46, 1.46)
CRP Score 2^cd^	0.86 (0.65, 1.13)	0.98 (0.69, 1.41)
Time to diagnosis ≤ 10 years	0.76 (0.47, 1.23)	1.11 (0.61, 2.03)
Time to diagnosis > 10 years	1.10 (0.73, 1.66)	0.93 (0.55, 1.56)
CRP Score 3^cd^	1.05 (0.76, 1.47)	1.07 (0.73, 1.59)
Time to diagnosis ≤ 10 years	1.01 (0.56, 1.81)	1.01 (0.46, 2.22)
Time to diagnosis > 10 years	1.16 (0.74, 1.83)	1.22 (0.69, 2.16)

Bold HR and CI values indicate statistical significance. Underline HR and CI values indicate borderline statistical significance after multiple comparison adjustment

^a^205 cases (3 cases with person-year = 0 or > 25 years were removed from the analytical dataset) Model adjusted for age at blood draw, sex, smoking status, BMI, surrogate variables for batch effects, and a pack-year-based smoking methylation score. The results for Inflammation Scores additional adjust for cell proportions. BMI was removed from model when stratified by BMI. Group person-year: all lung cancer cases = 421.3; cases with time to diagnosis ≤ 10 years = 104.3; cases with time to diagnosis > 10 years = 316.9; cases with BMI < 25 kg/m^2^ = 184.3; cases with BMI ≥ 25 kg/m^2^ = 236.9

^b^149 cases (1 case with person-year =  >25 years were removed from the analytical dataset) Model adjusted for age at blood draw, sex, smoking status, BMI, surrogate variables for batch effects, and methylation-predicted pack-years smoked. The results for Inflammation Scores additional adjust for cell proportions. BMI was removed from model when stratified by BMI. Group person-year for all non-small cell lung cancer (NSCLC) cases = 337.6; NSCLC cases with time to diagnosis ≤ 10 years = 84.4; NSCLC cases with time to diagnosis > 10 years = 253.2; NSCLC cases with BMI < 25 kg/m^2^ = 141.4; NSCLC cases with BMI ≥ 25 kg/m^2^ = 196.2

^c^HR results reported per 1 unit of SD increase among all cases (mdNLR SD: 1.86; CD4/CD8 ratio SD: 2.14; BL ratio SD: 0.0534; TL ratio SD: 0.0844; ML ratio SD: 0.125; NM/L ratio SD: 1.38; EL ratio SD: 0.079; CD4nv/L ratio SD: 0.0920; B/CD8 ratio SD: 2.21; CD8/Treg ratio SD: 112; Bnv/Bmem ratio SD: 81.3; CD4nv/CD4mem ratio SD: 0.335; CD8nv/CD8mem ratio SD: 1.64; CpG Score 1 SD: 0.979; CpG Score 2 SD: 0.945; CpG Score 3 SD: 1.02) and HR results reported for Treg > 0 versus Treg = 0 among all cases; Bonferroni-adjusted CI reported for all except mdNLR and CRP Scores 1–3 (a prior hypotheses)

^d^CpG Scores 1, 2, and 3 are built using 54 CpG sites, the top 10 highly cell-specific CpG sites, and the 10 modestly cell-specific CpG sites, respectively

We observed a 47% increased risk of dying for one standard deviation of mdNLR for NSCLC cases (*n* = 149 cases; HR: 1.47 [1.20, 1.81]). Among the NSCLC cases whose mdNLR was from <  = 10 years before their diagnosis, we found a 73% increased risk of dying for a one standard deviation increase in mdNLR (HR: 1.73 [1.19, 2.51]). In comparison, the risk of dying for a one standard deviation increase in mdNLR was lower among the NSCLC cases whose mdNLR was from 10 to 25 years prior to diagnosis (HR: 1.39 [1.05, 1.85]). Lastly, we observed a 28% increased risk of dying from lung cancer for one standard deviation increase in mdNLR (*n* = 205 cases deleted 3 cases with person-year = 0 or > 25 years; HR: 1.28 [1.09, 1.50]).

Immune cell ratios for CD4/CD8, NLR, B cell/lymphocyte, T cell/lymphocyte, Mono/lymphocyte, Eos/lymphocyte, CD4nv/lymphocyte, B cell/CD8, CD8/Treg, Bnv/Bmem, CD4nv/CD4mem, CD8nv/CD8mem, and Treg (> 0 vs = 0) were not associated with lung cancer-specific death, except for a 48% increased risk for one standard deviation increase in Neu + Mono/lymphocyte ratio among the NSCLC cases (HR: 1.48 [1.04, 2.11]) and a borderline significant 29% increased risk of dying from lung cancer (HR: 1.29, adjusted CI: [1.00, 1.67]) for one standard deviation increase in Neu + Mono/lymphocyte ratio after multiple comparison adjustments. Furthermore, the three CRP Scores were not associated with lung cancer-specific death.

## Discussion

Our study prospectively assessed predicted immune cell profiles using DNA methylation markers and examined associations between previously identified DNA methylation markers of inflammation and lung cancer risk and survival. Using pre-diagnostic blood samples of lung cancer cases and controls who participated in the CLUE I/II cohorts [23], pre-diagnosis mdNLR was associated with increased risk of NSCLC, and among cases, with total lung cancer and NSCLC lung cancer-specific death. In addition, we built a series of methylation-derived CRP scores to capture individual systemic inflammatory profiles years before lung cancer diagnosis; these scores were inversely associated with risk of lung cancer, especially for NSCLC after adjusting for methylation-predicted pack-years smoked, but not with lung cancer-specific mortality.

Studies on NLR (calculated from measured WBC differentials) and lung cancer risk and survival typically measure pre-treatment NLR at diagnosis or up to 30 days prior to treatment [28–30]. Unlike prior studies, we were able to assess individual systemic inflammation profiles many years prior to diagnosis by using methylation markers of inflammation. Our study is not directly comparable to prior studies since we measured mdNLR using blood samples from subjects with a median of 14 years prior to lung cancer diagnosis. In addition, most cases in our study were diagnosed before the widespread use of immunotherapy. To our knowledge, only one other cohort, the multicenter β-Carotene and Retinol Efficacy Trial (CARET), examined pre-diagnosis mdNLR and lung cancer risk and survival using blood drawn years prior to diagnosis (median 4.7 years) [31, 32]. CARET, a study of heavy smokers, reported a 21% increased risk of lung cancer per one unit increase in mdNLR (OR: 1.21 [1.01, 1.45]), a 30% increased risk of NSCLC for one unit increase in mdNLR (OR: 1.30 [1.03, 1.63], and no association between higher pre-diagnosis mdNLR and risk of developing SCLC (OR: 1.06 [0.77, 1.47]) [31]. Like in CARET, in CLUE I/II we observed a 47% increased risk of NSCLC for a one standard deviation increase in mdNLR (*n* = 150 pairs; OR: 1.47 [1.08, 2.02]), but in contrast to CARET, we found no statistically significant association for overall lung cancer risk.

CARET researchers recently reported that pre-diagnosis mdNLR was positively associated with increased mortality for SCLC cases, but not for other case types [32]. In comparison, we observed a positive association between pre-diagnosis mdNLR and lung cancer-specific and NSCLC-specific mortality. In the case of SCLC, the number of cases was too limited for us to estimate stable associations (*N* = 29). Taken together, the CLUE and CARET results suggest that a systemic inflammatory profile marked by elevated NLR could indicate a lesser ability to mount a robust immune response to a developing lung cancer and/or a more favorable environment for cancer progression. Differences in findings between the two studies could stem from differences in study populations. The CARET cohort is exclusively heavy smokers, including a subgroup exposed to asbestos. In comparison, our analysis in the CLUE I/II cohorts included never, ever, and current smokers. Furthermore, our study population had a lower mdNLR in the lung cancer cases (mean 1.86 and SD 1.32) than in CARET (mdNLR mean 2.18 and SD 1.46).

Using a newly expanded deconvolution library, we were able to parse apart the granulocyte subtypes (neutrophils, eosinophils, and basophils) and investigate the balance between naïve and memory cell compartments for lung cancer. Previous research has identified the monocyte/lymphocyte (or lymphocyte/monocyte) ratio as an independent prognostic factor in NSCLC, demonstrating significant association with overall survival in patients with NSCLC [33–35]. In comparison, our exploratory analyses of immune cell ratios suggest that one standard deviation increase in the monocyte/lymphocyte ratio could potentially indicate increased risk of NSCLC after additionally adjusting for methylation-predicted pack-years. In addition, we found an increased risk of dying from lung cancer associated with an increase in Neu + Mono/lymphocyte ratio among the NSCLC cases after multiple comparison adjustments.

We also investigated three CRP Scores that we built from 54 CpG sites that had been strongly associated with CRP in previous studies. We found these methylation-predicted CRP Scores to be moderately correlated with log-CRP and log-IL6 in the controls of a previously published pancreatic cancer dataset [27]. CRP is a systemic marker of chronic inflammation and has been reported as a risk factor for cancer development [36]. Previous studies of pre-diagnostic circulating CRP concentration and lung cancer risk (7 cohorts [10, 11, 19, 37–39] and 3 nested case–control studies [8, 12, 40]) have consistently found a moderate positive association between pre-diagnostic CRP concentrations and lung cancer risk. In our study, CRP Scores were not associated with lung risk when taking into account the matching factors, BMI, and batch effects. However, we observed an inverse association when additionally adjusting for methylation-predicted pack-year. Our results suggest that when strict control of smoking is applied, our CRP Score is likely capturing the unique individual immune response that is not driven by smoking.

Furthermore, these results provide preliminary evidence supporting the hypothesis that systemic inflammation not driven by smoking could have a protective effect on individuals. While smoking is by far the most important risk factor for lung cancer, our DNA methylation-based CRP Scores provide the opportunity to examine inflammatory measures not related to smoking that could play a role in modulating cancer risk years prior to diagnosis. Lastly, our experience with the CRP Scores suggests that measuring methylation-derived inflammatory responses using pre-diagnostic samples provides the opportunity to capture informative individual systemic inflammatory profiles years prior to diagnosis, potentially shedding light on risk factors key to lung cancer development and progression, e.g., underlying genetics, exposure to environmental risk factors, and behavior risk factors.

Like other observational studies, our study included a limited number of NSCLC and SCLC cases. The relatively small sample size of SCLC cases (*N* = 29) impacted our ability to observe associations for this subtype (SCLC comprises about 15% of lung cancer cases in the USA). In our survival analysis, we adjusted for stage and restricted our analysis to samples whose time between blood draw and date of lung cancer diagnosis was less than 25 years; however, our survival analysis did not have access to post-diagnosis smoking status information. Our study is also limited by a lack of replication dataset and reduced generalizability. (Study population is mainly White and with very few cases in never smokers.) The CRP Scores we built should be investigated in other populations to ensure that what we observed did not arise due to chance.

## Conclusions

Our study suggests that elevated pre-diagnosis mdNLR and a lower non-smoking-related systemic inflammatory profile before diagnosis are associated with higher cancer risk and poorer lung cancer-specific survival. These relationships were especially evident for NSCLC. As the most common subtype of lung cancer, most NSCLC cases are diagnosed with locally advanced or metastatic disease. Our prospective results support future evaluation of whether DNA methylation-based inflammatory measures could enhance lung cancer risk stratification to improve targeted lung cancer screening.

## Methods

### Study Population

This nested case–control study selected cases and controls from individuals who participated and provided blood in both CLUE I and CLUE II [26]. The CLUE I cohort was developed to identify serologic precursors of cancer and was conducted in Washington County, Maryland, in the fall of 1974. A blood sample was collected from 25,620 volunteers at the time of participation [41, 42]. The CLUE II cohort was conducted from May through October 1989. During this time, 32,894 participants donated a blood sample which was collected in tubes containing heparin and kept chilled until centrifuged, aliquoted into plasma, erythrocytes, and buffy coat, and frozen at 70 °C [43]. In CLUE II, the baseline for this study, health information was collected at the time of blood draw, including attained education, cigarette smoking status, cigarette smoking dose, cigar/pipe smoking status, and self-reported weight and height.

Incident lung cancer cases were ascertained from linkage to the Washington County cancer registry (before 1992 to the present) and the Maryland Cancer Registry (since 1992 when it began to the present). We ascertained 241 incident lung cancer cases who participated in CLUE I and were diagnosed after the day of blood draw in CLUE II through January 2018. Cases were characterized with respect to histology. We used incidence density sampling to select one control matched to each case on age, sex, smoking status and intensity (cig/day), and cigar/pipe smoking status. Death from lung cancer as the underlying cause was obtained from death certificates. The Institutional Review Board at the Johns Hopkins Bloomberg School of Public Health and the Tufts University Health Sciences Campus Institutional Review Board approved this study.

### DNA methylation measurements

Extracted DNA was bisulfite-treated using the EZ DNA Methylation Kit (Zymo), and DNA methylation was measured with the 850 K Illumina Infinium MethylationEPIC BeadChip Arrays (Illumina, Inc., CA, USA). All samples and all array experiments were performed blinded to case–control status. Details on DNA methylation measurements, data preprocessing processing, and quality control assessment/screening are provided in the Additional file 1. The 850 K methylation microarray has been validated from a biological and technical standpoint. Reproducibility of results from 850 K Illumina array has been previously shown to be very high (*r* = 0.997) [44]. DNA volume and quality were sufficient for 208 of the cases and 222 controls totaling 208 matched pairs.

### Estimation of peripheral blood leukocyte composition

Peripheral blood leukocyte subtypes proportions, including myeloid lineage sub-types [neutrophils (Neu), eosinophils (Eos), basophils (Bas), and monocytes (Mono)] and lymphoid lineage subtypes [B lymphocytes naïve (Bnv), B lymphocytes memory (Bmem), T helper lymphocytes naïve (CD4nv), T helper lymphocytes memory (CD4mem), T regulatory cells (Treg), T cytotoxic lymphocytes naïve (CD8nv), T cytotoxic lymphocytes memory (CD8mem), and natural killer lymphocytes (NK)], were estimated using a newly expanded reference-based deconvolution library EPIC IDOL-Ext [45]. This library used the IDOL methodology [46] to optimize the currently available six-cell reference library [47] in order to deconvolve the proportions of 12 leukocyte subtypes in peripheral blood. This EPIC IDOL-Ext library (Bioconductor package FlowSorted.BloodExtended.EPIC) was validated using flow cytometry gold standard data and substantiated by including publicly available data from > 100,000 samples [45].

### Methylation-Derived Neutrophil Lymphocyte Ratio (mdNLR)

The peripheral blood neutrophil-to-lymphocyte ratio (NLR) is a cytological marker of both inflammation and poor outcomes in cancer patients [48–52]. We used a DNA methylation-derived NLR (mdNLR) index to predict the common clinical NLR parameter using a previously described approach [9]. This index is based on normal isolated leukocyte reference DNA methylation libraries and established reference-based cell mixture deconvolution algorithms [9, 53].

### Inflammation-associated CpG score

We used 54 CpG sites that have been strongly associated with C-reactive protein (CRP) [54, 55] to build three CRP Scores. We selected these 54 CpGs (remaining 4 were not on the 850 K array that we used) from the 58 CpGs identified by Ligthart and colleagues [54] for their association with serum CRP level (listed in Table 3) using 450 K DNA methylation data. Forty-five of these 58 CpG sites were validated to have the same direction of protein–methylation associations by Myte et al. [55]. These CpGs, while identified based on their CRP association, have also been shown to be associated with other inflammatory mediators [54–56]. To compute CRP Score 1, we multiplied the beta value at each selected CpG site with the effect size estimates reported by Ligthart et al. These estimated beta coefficients represented the change in DNA methylation per one unit increase in log CRP. In the CRP Score 1 formula, we weighted the beta coefficients estimated by Ligthart et al. with their corresponding standard errors.

$${\text{CRP}}\;{\text{Score}}_{i} = \sum B_{ij} \times \frac{{\Delta_{j} }}{{{\text{SE}}_{j} }}$$

*B*_*ij*_ is the beta value for the *i*th participant at the *j*th CpG site. ∆_*j*_ is the beta coefficients reported by Ligthart et al. for the *j*th CpG site. *SE*_*j*_ is the SE reported by Ligthart et al. for the *j*th CpG site.

Since most of the estimated beta coefficients are negative, CRP Score 1 ranged between − 0.059 and -0.026 in these participants. A score closer to zero indicated higher CRP levels. Based on CRP Score 1, we computed two additional CRP Scores, one cell (leukocyte)-type invariant (CRP Score 2) and one cell-specific (CRP Score 3). Among the 54 inflammation (CRP)-associated CpGs, we identified putative cell-type invariant and cell-specific CpGs by conducting ANOVA using the dataset described in Salas and Koestler et al. [47] and publicly available on the Gene Expression Omnibus (GSE110555). The dataset used for this ANOVA consisted of EPIC methylation data profiled in purified leukocyte cell population isolated from different healthy adults. Specifically, methylation signatures were available for CD4 + T cells, CD8 + T cells, NK cells, B cells, monocytes, and neutrophils. One-way ANOVA models were fit independently to each of the 54 CRP-associated CpGs treating methylation as the dependent variable and cell type as the independent variable. We tested the null hypothesis that the mean methylation beta-value is the same across the cell types. The *F*-statistic, corresponding *p* value, and maximum absolute pairwise difference in the mean methylation beta value across cell types were calculated for each of the 54 CpGs. We then selected subgroups of CpG sites that had the top 10 smallest or top 10 largest *F*-statistic value to build the two additional CRP Scores. CRP Score 2 consists of putative cell-specific CpGs with high *F*-statistics, e.g., those exhibiting a difference in mean methylation beta-values between at least two of the six cell types. CRP Score 3 is made of cell-type invariant CpGs with low *F*-statistics, e.g., CpGs for which there did not appear to be a substantial difference in mean methylation beta-values across the normal six leukocyte subtypes. Score 2 ranged between − 0.0002 and 0.0046, while Score 3 ranged between − 0.025 and − 0.016. In the regression analyses, we used a standardized version of CRP Scores 1, 2, and 3 (mean = 0, sd = 1) for easier interpretation of results and allowing us to compare the results for each of the scores.

### Statistical analyses

All statistical analyses were performed in R (version 3.5.1). We estimated mdNLR as described above, used an independent pancreatic cancer dataset [27] to estimate the correlation between estimated values of CRP Scores 1–3 with the log CRP and log IL-6 levels, and tested a series of a priori hypotheses concerning the mdNLR and CRP Scores. In addition, we also conducted exploratory analyses to generate novel hypotheses regarding the role of methylation-derived leukocyte proportions in lung cancer. Immune cell ratios (e.g., CD4/CD8, Neu/lymphocyte, B cell/lymphocyte, T cell/lymphocyte, Mono/lymphocyte, Neu + Mono/lymphocyte, Eos/lymphocyte, CD4nv/lymphocyte, B cell/CD8, CD8/Treg, Bnv/Bmem, CD4nv/CD4mem, and CD8nv/CDmem) were calculated for each sample by taking the ratio of its predicted cell proportions described above and tested as continuous variables. The presence of Treg was tested as a dichotomous variable. Given the need for multiple comparison adjustment, Bonferroni adjustment (family-wise error rate = 0.0013) was conducted for all exploratory analyses.

We used conditional logistic regression to examine the association between DNA methylation-based inflammatory measures (CRP Scores 1–3 and continuous mdNLR) and lung cancer risk. Models were fit with age, sex, and smoking status (never, former, current) as matching factors and were adjusted for potential confounding factors, including body mass index (BMI), batch effect, and previously described methylation-predicted pack-years smoked [57]. These analyses did not additionally adjust for methylation-derived cell proportions given how these proportions correlated with methylation-based inflammatory measures (Table 3). We repeated these analyses by lung cancer histology (NSCLC, SCLC), length of time between blood draw and diagnosis (< = 10, > 10 years), and BMI (< 25, ≥ 25 kg/m^2^).

Among the lung cancer cases, we examined the association between these same pre-diagnostic DNA methylation-based inflammatory measures (CRP Scores 1–3 and continuous mdNLR) and risk of lung cancer-specific death using a series of multivariable Cox proportional hazard regression adjusting for age, gender, smoking status, BMI, stage at diagnosis (three strata: stage 1 & 2, stage 3 & 4, and missing), cell proportion, batch effects, and methylation-predicted pack-years smoked. The proportional hazards assumption was checked by conducting global tests of correlating the set of scaled Schoenfeld residuals with time for each covariate. We excluded three lung cancer cases whose date of diagnosis and date of death were the same, or whose time between blood draw and date of lung cancer diagnosis was longer than 25 years. Cases were followed until their date of death from lung cancer, death from another cause, or the end of follow up in 2018, whichever came first.

## Supplementary Information

**Additional file 1**. Supplementary Methods.

## Data Availability

The datasets generated during the current study are available from the corresponding author on reasonable request and will be deposited into dbGaP by publication.
